# Heat-Stable Enterotoxin Secretions Assessed via ICP-MS Reveal Iron-Mediated Regulation of Virulence in CFA/I- and CS6-Expressing ETEC Isolates

**DOI:** 10.3390/cells12040567

**Published:** 2023-02-10

**Authors:** Ian E. Hollifield, Natalya I. Motyka, Sydney R. Stewart, Michelle D. Blyth, Kaylynn A. Fernando, Kristen L. Clement, Jacob P. Bitoun

**Affiliations:** Department of Microbiology and Immunology, Tulane University School of Medicine, New Orleans, LA 70112, USA

**Keywords:** enterotoxins, diarrhea, ETEC, secretion, metals, adhesin

## Abstract

Enterotoxigenic *Escherichia coli* (ETEC) are a significant cause of childhood diarrhea in low-resource settings. ETEC are defined by the production of heat-stable enterotoxin (ST) and/or heat-labile enterotoxin (LT), which alter intracellular cyclic nucleotide signaling and cause the secretion of water and electrolytes into the intestinal lumen. ETEC take cues from chemicals (e.g., glycans, bile salts, and solutes) that may be liberated following enterotoxin activity to recognize entrance into the host. ETEC then alter the expression of surface adhesins called colonization factors (CFs) to attach to the intestinal epithelium, proliferate, and cause disease. Here, we used an in vivo model of oral ST intoxication to determine its impact on luminal ion concentrations via ICP-MS. We also used functional assays, including Western blots, qPCR, and toxin activity assays, to assess the impact of luminal ion flux on CF and toxin expression. Finally, we assessed ETEC strains with CFs CFA/I or CS6 in a streptomycin mouse model of ETEC colonization. ST causes rapid and significant increases in luminal chloride but significant decreases in luminal magnesium and iron. We confirmed that increased sodium chloride suppresses CFA/I production in ETEC H10407 but does not affect CS6 production in ETEC 214-4. CFA/I production in ETEC H10407 is increased when magnesium becomes limiting, although it does not affect CS6 production in ETEC 214-4. Iron restriction via deferoxamine induces CFA/I expression in ETEC H10407 but not CS6 expression in ETEC 214-4. We demonstrate that ST production is suppressed via iron restriction in H10407, 214-4, and over 50 other ETEC clinical isolates. Lastly, we demonstrate that the iron restriction of mice using oral deferoxamine pre-treatment extends the duration of ETEC H10407 (CFA/I^+^) fecal shedding while accelerating ETEC 214-4 (CS6^+^) fecal shedding. Combined, these data suggest that enterotoxins modulate luminal ion flux to influence ETEC virulence including toxin and CF production.

## 1. Introduction

Enterotoxigenic *Escherichia coli* (ETEC) are prominent causes of diarrheal disease that particularly affect children in low-to-middle income countries (LMICs) [1]. Recent epidemiological data also demonstrate that ETEC can be carried asymptomatically or oligosymptomatically in children and adults [2,3], and they may contribute to other co-morbidities, including growth faltering or intellectual stunting later in life [4]. Although the molecular details that cause these co-morbidities have not been elucidated, they are almost assuredly related to repeated exposure to heat-stable (ST) and/or heat-labile (LT) enterotoxins.

ETEC are introduced via the fecal-oral route and use adhesins called colonization factors (CFs), which can be fimbrial, fibrillar, or afibrillar, to attach to the small intestinal epithelium [5], where the enterotoxins induce secretory diarrhea. Over 30 different antigenic CFs have been identified, although some of their binding partners remain elusive. Furthermore, the Global Enteric Multicenter Study (GEMS) demonstrated that many ETEC isolates do not encode any recognizable CFs, while other ETEC isolates express more than one CF [6]. Major CFs include class 5 fimbriae CFA/I and coli surface (CS) antigens CS1-CS6. In children, antibodies against CFA/I are found following natural infection and negatively correlate to the risk of CFA/I-ETEC infection [7]. Recent studies have demonstrated that human volunteers prophylactically treated with hyperimmune bovine colostrum rich in antibodies against CS6 are not protected in an ETEC challenge model [8]. Since CFA/I and CS6 are two of the most predominant ETEC CFs, and antibodies against CFA/I, CS6, and LT protect against over 50% of all ETEC isolates, CFs remain attractive vaccine targets [9].

ETEC H10407 was isolated from an episode of secretory diarrheal disease in Bangladesh in the 1970s [10]. Since then, it has become the most well-studied ETEC isolate and expresses the CFA/I adhesin, LT, and both porcine and human versions of ST (STp and STh). Comparatively, ETEC 214-4 was isolated as the etiological agent of secretory diarrheal disease in travelers to Mexico and was found to express CS6 adhesin and STp [11]. Both ETEC H10407 and ETEC 214-4 have been used successfully in controlled human ETEC infection models [12,13]. Our previous in vitro studies demonstrated that ST production varies among ETEC strains [14], suggesting that ST-mediated secretory diarrhea severity could potentially be modulated by other virulence (toxins, adhesins, chaperones), environmental (oxygen tension, pH, redox potential, ions, microbiome), or host factors.

Enterotoxin activity ultimately induces the accumulation of second messenger cyclic nucleotide signals. Specifically, ST induces cGMP, and LT induces cAMP. Subsequent signaling cascades via protein kinase G (PKG) or protein kinase A (PKA) lead to the accumulation of electrolytes (e.g., chloride and sodium ions) and water into the intestinal lumen (e.g., secretory diarrhea). LT provides ETEC with a colonization advantage [15,16], but the prevailing dogma for why ETEC express enterotoxins is that they provide a dissemination advantage over closely related species. However, it also remains possible that ETEC encode enterotoxins to modulate the concentrations of key nutrients or ions in the luminal microenvironment to favor their pathogenesis. Increased osmolality down-regulates fimbriae expression in certain pathogenic and non-pathogenic *E. coli* [17], suggesting that CF expression in ETEC may also be regulated by enterotoxin-mediated ion flux across the intestinal epithelial membrane. 

Iron acquisition is a longstanding virulence factor for enteric pathogens, and it remains plausible that local iron availability regulates the development of innate and adaptive immunity to ETEC. Our previous studies demonstrated that ST binds iron and zinc under reductive conditions [18], and we speculated that ST may shuffle between a toxin (oxidized version) and a siderophore (reduced version) depending on intraluminal redox potential. This was supported by experiments showing that iron limitation in chemically defined media caused increased ST antigenicity in ETEC culture supernatants but decreased cGMP production, as compared to supernatants from ETEC grown in the presence of iron. Iron represses CFA/I expression in ETEC-type strain H10407 [19], and deferoxamine, a hydroxamate siderophore, increases CFA/I expression in ETEC H10407 [20]. On the other hand, ferrous iron salts increase CS6 expression and adherence to intestinal monolayers in vitro [21]. Thus, ETEC adherence and dissemination may be regulated by enterotoxin-mediated fluxes in host ions and the ion-microbiome-immune axis, of which iron may be a key factor in disease outcomes. 

ETEC subvert the development of long-lasting immune responses [22,23], but the role of enterotoxin-mediated flux in luminal ions and iron restriction on ETEC infection remains unknown. This is especially important since ETEC are diverse. The purpose of this study was to delineate some of the major ions secreted into and out of the intestinal lumen following ST intoxication to elucidate how those ions affect ST and CF production in CFA/I^+^ and CS6^+^ ETEC isolates.

## 2. Materials and Methods

### 2.1. Bacterial Strains and Culture Conditions

All ETEC isolates used in these studies were obtained from previously archived collections [6,14] and can be found in Table S1. Spontaneous streptomycin-resistant variants of H10407 and 214-4 were generated by repeatedly passaging colonies from the original stocks onto LB agar plates with increasing streptomycin concentrations. ETEC were streaked from glycerol stocks onto LB or CFA agar [24]. ETEC were then subcultured 1:100 into Erylenmeyer flasks with CFA media, LB media, or 4AA media [25]. Note that 4AA minimal media is a chemically defined media (12.3 mM proline, 6.61 mM aspartic acid, 6.3 mM serine, 4.5 mM alanine, 50 mM K_2_PO_4_, 42.8 mM NaCl, 18.7 mM NH_4_Cl, 5.6 mM Tricine, 1.4 mM Na_2_SO_4_, 0.05% sodium lactate, 246 µM MgCl_2_, 31 µM FeCl_3_, and 25 µM MnCl_2_) that was used for most of our experiments. In some cases, deferoxamine mesylate was obtained from Sigma and added to 4AA media before ETEC inoculation. Following culturing and subculturing, the bacterial cultures were harvested via centrifugation for 5 min at 14,000 rpm. Cell-free supernatants were retained to examine ST production and ST activity via cGMP measurements. ETEC pellets were resuspended in PBS and lysed via sonication with a Sonifer Cell Disruptor W185 for ten 2-second pulses, then clarified by centrifugation at 14,000 rpm for 30 min. Supernatants and lysates were then assessed for protein content by Pierce BCA (Thermo Scientific 23225, Waltham, MA, USA). 

### 2.2. ST ELISAs

Competitive ST enzyme-linked immunosorbent assays (ELISAs) with monoclonal anti-ST antibodies were carried out similarly to what has been previously described [26]. ELISA coating antigens were prepared via glutaraldehyde conjugation of STh to ovalbumin. Microtiter plates were coated overnight with 0.1 µg per well of ST-Ova conjugate in ELISA buffer (128 mM NaCl, 2.7 mM KCl, 1.5 mM KH_2_PO_4_, and 8.1 mM Na_2_HPO_4_, pH 7.2). Cell-free supernatants from ETEC cultures were pre-incubated with a monoclonal ST antibody (Fitzgerald M120530) at a 1:16,000 dilution before being added at 1:2 dilutions down respective columns of the ST-Ova coated plate. They were then incubated for two hours at room temperature before washing the plates and incubating with rabbit anti-mouse IgG-AKP conjugate (Sigma A1902, St. Louis, MO, USA), which was used as a secondary antibody at a 1:400 dilution for 1 h at room temperature. Plates were developed for 30 min to 1 h with 0.1 mL of freshly prepared developing buffer (1 mg/mL *para*-nitrophenolphosphate (Sigma, N2765) dissolved in 9.7% diethanolamine, 0.5 mM MgCl_2_, pH 9.8). Absorbance at 405 nm was read using a Bio-Tek plate reader.

### 2.3. ST Activity Assays

Human T84 colonic epithelial cells (ATCC CCL-248) were cultured regularly in Dulbecco’s modified Eagle’s medium (DMEM) supplemented with 5% fetal bovine serum (FBS). To assess ST activity, confluent T84 cells seeded in a 24-well plate were washed with sterile PBS, then given fresh DMEM with 1% FBS supplemented with 30 µM vardenafil (Cayman, 9001800) and 20 µM zardaverine (Tocris Bioscience, 1046, Bristol, UK). After a one-hour preincubation with these phosphodiesterase inhibitors, T84 cells were treated with purified toxins (controls) or mass-calibrated cell-free supernatants from ETEC isolates for 2 h. T84 cells were then lysed with a cell lysis buffer (1% Triton X-100, 0.5% sodium deoxycholate, 0.1% SDS, 150 mM NaCl, 50 mM Tris-HCl pH 8.0), and lysates were diluted 1:200 and tested for cGMP content using cGMP ELISA kit (Cayman 581021) according to the manufacturer’s instructions.

### 2.4. Western Blots

Purified CFA/I (NR-49109) and CS6 (NR-49114) were obtained from BEI Resources and used as positive controls for Western blots. Clarified ETEC lysates were loaded at equal mass onto a 10% polyacrylamide gel and run at 160 V for 35 min, then transferred to a nitrocellulose membrane using the iBlot 2. The membrane was blocked with 5% skim milk in 0.05% PBST, then probed with rabbit antisera against CFA/I (1:3000) or CS6 (1:1000) (courtesy of Dr. Eileen Barry, University of Maryland). The secondary antibody (1:2500) was a goat-produced anti-rabbit IgG-HRP conjugate (Southern Biotech 1040-05, Birmingham, AL, USA) developed using a peroxidase substrate solution (Thermo Scientific 32209). The Western blots were imaged using an Amersham Imager 600, and post-image densitometry analysis was carried out using ImageJ (version 1.53m).

### 2.5. Patent Mouse Model

The adult patent mouse assays (PMAs) were carried out as previously described [23,27]. In brief, 6–10-week-old female BALB/c mice were fasted overnight while allowed water ad libitum and orally gavaged the following morning with saline (*n* = 3) or ST (25 µg) (*n* = 3). Mice were sacrificed via CO_2_ asphyxiation and cervical dislocation 30 min, 60 min, or 180 min post-gavage, as previously described [28]. The gut-to-carcass ratio was measured, and luminal secretions were collected. 

### 2.6. Elemental Analysis

Inductively coupled plasma mass spectroscopy (ICP-MS) was carried out at Tulane University using an Element2 high-resolution ICP-MS. Luminal secretions were diluted 10-fold into Hyclone water before heating to 60 °C for 3 h in the presence of 1% hydrogen peroxide. After incubation, the samples were centrifuged for 10 min at 14,000 rpm and then added to new tubes containing 4% nitric acid. The samples were incubated overnight at 60 °C. After incubation, the samples were centrifuged for 10 min at 14,000 rpm and then diluted 2-fold to a final concentration of 2% nitric acid. The samples were again incubated overnight at 60 °C. Next, the samples were centrifuged for 10 min at 14,000 rpm and then sterile-filtered using 0.2 µM filters. Samples were then diluted 1:10 and introduced via a FAST autosampler using a PC3-cooled spray chamber. Ca(44), Fe(56), Mg(24), Mn(55) and Zn(66) were analyzed in medium mass resolution, and a separate analysis was performed for Cl(35) using medium mass resolution. Calibration curves were constructed from stock standards. Cl was from 0.1–50 ppb. Mg was from 1–100 ppb. Ca was from 5–100 ppb, and Mn and Zn were from 0.25–50 ppb. Fe was from 0.5–100 ppb. Concentrations of each element in the luminal secretions were calculated based on interpolation of the linear fit curve by forcing data through zero. 

### 2.7. Real-Time PCR

For qPCR studies, bacteria were streaked from glycerol stocks onto LB plates; then, single colonies were cultured in 4AA broth. Overnight cultures were diluted 100-fold into fresh 4AA broth with or without deferoxamine (100 µM) and harvested at an OD_600_ of 0.5. Cells were treated with RNAprotect (Qiagen 1018380, Hilden, Germany); then, RNA was purified via repeated phenol-chloroform extraction. The resulting nucleic acid precipitate was resuspended in nuclease-free H_2_O, digested with DNAse I (Ambion AM2222, Austin, TX, USA), and purified using Qiagen’s RNeasy Plus kit (Qiagen 74134). cDNA was prepared using 250 ng–1000 ng RNA via iScript kit (BioRad 1708891, Hercules, CA, USA) before being diluted at 1:40 for qPCR analysis. qPCR reactions were carried out using iTaq Universal SYBR Green Supermix (BioRad 1725121) on a CFX Connect according to the manufacturer’s protocol. Fold-change was determined via the ΔΔCt method, whereby each gene of interest was calibrated to the 16S ribosomal housekeeping gene. Primers used for qPCR analysis can be found in Supplemental Table S2.

### 2.8. Streptomycin Water ETEC Infection Model

The streptomycin water ETEC colonization model was carried out as described previously [29]. Female CD1 mice aged 7–9 weeks from Charles River Laboratories were used for the ETEC colonization model. Upon arrival, mice were allowed to adapt to their housing for a minimum of 72 h before experimental intervention. Streptomycin (5 g/L) was provided in the drinking water 48 h before infection and withdrawn 24 h later. Food was withdrawn 18 h before infection. Cimetidine (1.0 mg) was administered intraperitoneally 1 h before infection. Deferoxamine-treated mice were given 0.6 mg deferoxamine orally 24 h before infection and again immediately before infection. Mice were orally infected with 10^8^ CFU of appropriate inoculum strain. Weight change and fecal shedding of bacteria were monitored daily. 

### 2.9. Statistical Analysis

All statistical analyses were performed using Prism 9 software (GraphPad, Inc., San Diego, CA, USA). Unpaired *t*-tests were used to analyze experiments containing two groups. In experiments with more than two groups, statistical analysis was performed using unpaired one-way analysis of variance (ANOVA), followed by Tukey’s or Bonferroni’s post hoc analysis as appropriate. *p* values are described in the figure legends. 

## 3. Results

### 3.1. ST Intoxication Causes Luminal Ion Flux That Affects ETEC Virulence Factor Expression

The window to capture ST-mediated luminal fluid accumulation is short since ST is a fast-acting toxin, but it is highly reproducible [18,23,27]. Our previous studies demonstrate that ST-mediated luminal fluid accumulation is best observed 30 min following oral intoxication [27], but we have observed robust ST-mediated luminal fluid accumulation in adult mice in as little as 15 min following oral intoxication (data not shown). It is important to note that our patent mouse model of ST enterotoxicity is not a diarrhea model *per se* since ST-mediated luminal fluid is reabsorbed before it makes its way out of the gut [27]. This phenomenon has led some to mistakenly classify ST as non-secretory [30]. Despite this, large-scale epidemiological data have shown that ST-ETEC drive more severe secretory diarrheal disease as compared to LT-ETEC [6]. Together, this suggests that ETEC must sustain ST production throughout infection or that virulence, environmental, or host factors, including ions, may help sustain ST activity. 

**Figure 1:**
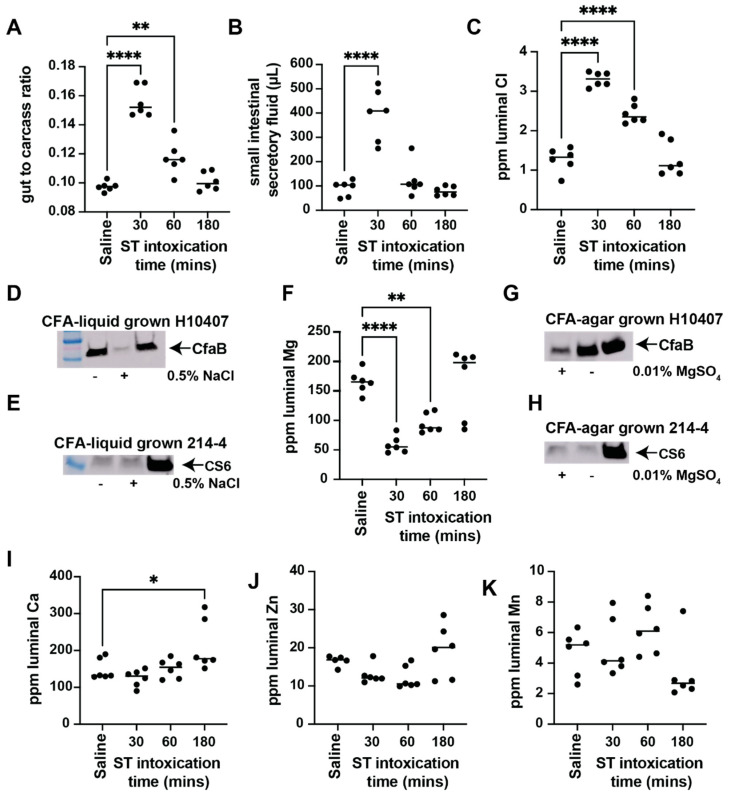
ST changes luminal Cl, Mg, and Ca content and differentially alters CF expression on ETEC H10407 and 214-4. Six-to-ten-week-old female Balb/c mice (*n* = 3 per group) were subjected to a patent mouse assay. The mice were gavaged with either saline or ST (25 µg). The saline-gavaged group was euthanized 30 min following oral gavage and served as the control group. The ST-gavaged groups were euthanized 30, 60, or 180 min following oral gavage. ST enterotoxicity, as measured by gut-to-carcass ratio, is evident 30 and 60 min following oral ST gavage (**A**). ST-mediated luminal fluid accumulation is significant after 30 min as measured by small intestinal luminal fluid recovered (**B**). ST intoxication causes a transient increase and flux in luminal chlorine (Cl) that peaks 30 min following intoxication before returning to baseline 180 min following intoxication (**C**). Exogenously added NaCl (0.5%) causes suppression of CFA/I production in ETEC H10407 (**D**) but does not affect CS6 expression in ETEC 214-4 (**E**). ST intoxication causes a transient decrease and flux in luminal magnesium (Mg) with a maximum drop 30 min following intoxication before returning to baseline 180 min following intoxication (**F**). CFA agar was prepared with and without MgSO_4_ (0.01%). Elimination of magnesium sulfate induces CFA/I production in ETEC H10407 (**G**) but does not affect CS6 expression in ETEC 214-4 (**H**). ST intoxication causes a significant increase in luminal calcium (Ca) 180 min following ST gavage, as compared to saline-gavaged controls (**I**). Luminal zinc (**J**) and manganese (**K**) were unchanged following ST intoxication, as compared to saline-gavaged controls. The murine studies and luminal ion analysis are shown as aggregated data from 2 experiments with 2–3 mice per experiment, thus displaying 5–6 data points per plot. Western blot experiments were verified in duplicate. Data were analyzed via one-way ANOVA. *, *p* < 0.05; **, *p* < 0.01; ****, *p* < 0.0001.

Using our standard patent mouse assay, we orally gavaged female Balb/c mice with saline (*n* = 3) or ST (*n* = 9). The ST-gavaged animals were then parsed into 30-min (*n* = 3), 60-min (*n* = 3), or 180-min (*n* = 3) ST intoxications. Similar to our previous studies, ST-mediated fluid accumulation is rapid and peaks 30 min following oral intoxication before waning (Figure 1A) [18,27]. Since we were interested in understanding the impact on luminal ions following ST intoxication, we collected the secretory contents contained within the small intestines. Small intestinal secretory fluid volume directly corresponds to ST enterotoxicity, and we measured significant differences in the volume of secretory contents following 30-min intoxication (Figure 1B). Secretory contents were then prepared for elemental analysis via inductively coupled plasma mass spectroscopy (ICP-MS, Tulane University, Shared Instrumentation Facility). We screened the luminal secretions for ions that may play a role in ETEC pathogenesis, including chloride, magnesium, calcium, manganese, zinc, and iron. Chloride is one of the major non-metal solutes secreted by intestinal epithelial cells via the activity of cystic fibrosis transmembrane regulator (CFTR) during secretory diarrheal disease [31] and is expected to be significantly elevated in the gut lumen during active secretion [32]. Previous studies using Ussing chambers have shown chloride efflux from intestinal tissues when stimulated with ST ex vivo [33]. Compared to saline-gavaged animals, our ICP-MS data show that luminal chloride is significantly elevated in animals gavaged with ST for 30 or 60 min (Figure 1C), validating this novel approach for the assessment of luminal ion changes in the patent mouse model. However, luminal chloride concentrations return to baseline levels, similar to those found within saline-gavaged controls, following ST intoxication for 180 min (Figure 1C). Although we did not screen for sodium ions, it is expected that luminal sodium would also be elevated during active secretion since cyclic nucleotide signaling blocks sodium proton exchanger 3 (NHE3) activity [34], resulting in the inhibition of epithelial sodium import. 

ETEC are highly adaptable to environmental conditions, and culturing methods contribute to increased or decreased virulence. For example, ETEC inoculums used for controlled human infection models are cultured on CFA agar [24], which is known to increase the expression of CFA/I fimbriae and increase attachment to human intestinal mucosa. Consistent with previous observations via hemagglutination assays [17], we demonstrate that 0.5% NaCl supplementation to liquid CFA media (a modification of CFA agar) decreases CFA/I production in ETEC H10407 (Figure 1D). ImageJ densitometry analysis demonstrates that the addition of 0.5% NaCl suppresses CFA/I production, as detected via Western blot, greater than 10-fold in ETEC H10407 when grown in CFA broth (Figure 1D). It is important to note that the addition of 0.5% NaCl did not impact the growth of ETEC H10407. This suggests that ST-mediated increases in luminal chloride could serve as cues to regulate ETEC attachment and subsequent pathogenesis. On the other hand, we found that the addition of 0.5% NaCl does not affect CS6 expression in ETEC 214-4 (Figure 1E) under similar conditions. This is not surprising since these CFs are encoded by different operons and have different promoter elements driving expression. However, these data highlight a problem with ETEC pathogenesis in that high genetic heterogeneity makes it difficult to identify overarching signals that affect virulence gene expression among diverse ETEC isolates.

Next, we report that magnesium concentrations are significantly decreased in luminal contents following 30 and 60 min of ST intoxication, as compared to saline-gavaged controls (Figure 1F). Luminal magnesium concentrations are restored to basal levels 180 min following ST intoxication (Figure 1F). This suggests that luminal magnesium levels drop during active secretion before returning to baseline. Standard CFA agar contains magnesium as a supplement. Therefore, we prepared two batches of CFA agar, one as defined (+magnesium) and one without the addition of 0.01% magnesium sulfate (−magnesium). Surprisingly, we demonstrate that CFA/I is induced when ETEC H10407 is cultured on magnesium-free CFA agar, as compared to magnesium-containing CFA agar (Figure 1G). ImageJ densitometry measurements reveal that 2.78-fold more CFA/I is produced when magnesium sulfate is withheld from the CFA agar medium (Figure 1G). This suggests that the ST-mediated decrease in luminal magnesium opposes the ST-mediated increase in luminal chloride to modulate CFA/I expression in ETEC H10407. On the other hand, we demonstrate that CS6 expression in ETEC 214-4 is not affected by magnesium concentrations (Figure 1H).

Calcium is an important intestinal signaling molecule and is required for the proper maintenance of adherens and tight junction proteins in intestinal epithelial cells [35]. Here we show that luminal calcium is significantly (*p* < 0.05) elevated 180 min following ST intoxication, as compared to saline controls (Figure 1I). Our data demonstrate that neither luminal manganese nor luminal zinc concentrations are changed following ST intoxication (Figure 1J,K) under the conditions studied.

**Figure 2:**
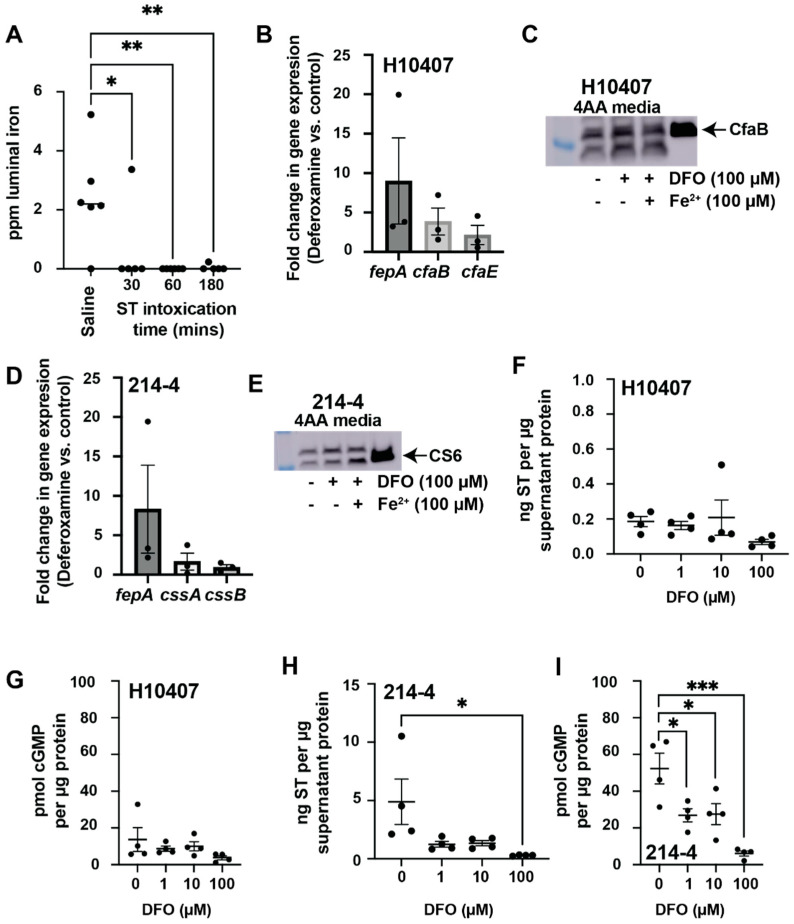
ST-induced depletion of luminal iron alters ST activity in ETEC H10407 and 214-4. ST intoxication causes a sustained decrease and flux in luminal iron (Fe) that begins during peak secretion (30 min of intoxication) and extends beyond 180 min (**A**). Iron limitation via deferoxamine mesylate (100 µM) increases expression of fepA, cfaB, and cfaE in ETEC H10407 (**B**). ETEC H10407 expresses more CFA/I when cultured with deferoxamine mesylate (100 µM) in 4AA media (**C**). Iron limitation via deferoxamine mesylate (100 µM) increases expression of fepA but not cssA and cssB in ETEC 214-4 (**D**). ETEC 214-4 expresses more CS6 when cultured with exogenous ferrous ammonium sulfate (100 µM) in 4AA media (**E**). ST production and secretion in ETEC H10407 cultured in 4AA media are unchanged in the absence and presence of increasing amounts of deferoxamine mesylate (1.0, 10, 100 µM) (**F**). No significant differences were observed when supernatants from ETEC H10407 cultured in 4AA media in the absence and presence of increasing amounts of deferoxamine mesylate (1.0, 10, 100 µM) were applied to T84 cells for ST-mediated induction of cGMP (**G**). ST production and secretion in ETEC 214-4 are suppressed when cultured in 4AA media containing deferoxamine mesylate (100 µM) (**H**). ST activity from ETEC 214-4 is significantly reduced when cultured in 4AA media containing deferoxamine mesylate (1.0, 10, 100 µM) (**I**). Data were analyzed via one-way ANOVA with matched conditions. *, *p* < 0.05; **, *p* < 0.01; ***, *p* < 0.001.

Most intriguingly, ST intoxication for 30, 60, or 180 min caused sustained depletion of luminal iron as compared to saline-gavaged controls (Figure 2A). Furthermore, our previous RNAseq data of ST-intoxicated T84 colonic epithelial cells demonstrated that the ST-induced transcriptome was similar to the transcriptome induced following deferoxamine administration [23,36]. Previous studies have shown that iron limitation via the use of iron chelator deferoxamine mesylate increases the *cfaE* expression in ETEC H10407 when grown aerobically in CFA broth [20]. CfaE is the tip adhesin of CFA/I that binds to multiple subunits of the stalk protein, CfaB. Here, using mid-log cultures of ETEC H10407 cultured in 4AA/lactate broth, we validate that iron limitation via deferoxamine (100 µM) significantly induces *cfaB* expression 3.87-fold and *cfaE* expression 2.16-fold (Figure 2B). We also confirmed that *fepA,* encoding for the ferrienterobactin receptor, was induced 9.00-fold following deferoxamine-mediated iron limitation (Figure 2B), as expected [37]. Next, we compared the relative abundance of CFA/I from ETEC H10407 lysates grown in 4AA broth (1) without deferoxamine, (2) with deferoxamine (100 µM), and (3) with deferoxamine (100 µM) and iron sulfate (100 µM). In accordance with increased transcript levels for *cfaE* and *cfaB*, we found that deferoxamine increases CFA/I expression in ETEC H10407 (Figure 2C). ImageJ densitometry measurements revealed that 1.43-fold more CFA/I is produced when deferoxamine is added to the 4AA broth. Furthermore, the addition of exogenous iron suppresses deferoxamine-mediated increases in CFA/I expression (Figure 2C).

Next, we explored how iron limitation via deferoxamine affects ETEC 214-4 virulence. Two transcripts (*cssB* and *cssA*) encode the functional CS6 adhesin of ETEC 214-4, and previous studies have demonstrated that CS6 is induced by increased iron levels [21]. Using mid-log cultures of ETEC 214-4 cultured in 4AA, we demonstrate that iron limitation via deferoxamine (100 µM) did not significantly induce the transcription of *cssB* and *cssA* as compared to controls (Figure 2D). The expression of *fepA* in ETEC 214-4 was induced 8.31-fold on average in deferoxamine-treated cultures compared to controls (Figure 2D), as expected. Next, we compared the relative abundance of CS6 from ETEC 214-4 lysates grown in 4AA broth (1) without deferoxamine, (2) with deferoxamine (100 µM), and (3) with deferoxamine (100 µM) and iron sulfate (100 µM). Surprisingly, ImageJ analysis shows that 1.54-fold more CS6 is produced when ETEC 214-4 is cultured with deferoxamine (Figure 2E). However, when ETEC 214-4 is cultured with both deferoxamine and exogenous ferrous iron, ImageJ analysis demonstrates that CS6 expression increases 2.68-fold greater than 4AA broth alone (Figure 2E). These data support previous studies demonstrating that iron increases CS6 expression [21]. Together, these data suggest that iron positively regulates CS6 expression in ETEC 214-4, while iron limitation positively regulates CFA/I expression in ETEC H10407.

We previously demonstrated that ETEC 214-4 produces and secretes more ST than ETEC H10407 [14]. Here we examined how iron limitation via deferoxamine impacts secreted ST production and activity in these isolates. In accordance with our previous studies, we show that ETEC H10407 makes ST, but there is no significant difference in ST production as measured via the ST competitive ELISA (Figure 2F) nor in the ST-mediated induction of cGMP in T84 cells (Figure 2G) when cultured in 4AA broth with increasing concentrations of deferoxamine (1, 10, 100 µM). On the other hand, cell-free supernatants from ETEC 214-4 analyzed via the ST competitive ELISA demonstrate that deferoxamine (100 µM) significantly suppresses ST production (Figure 2H) and ST activity as measured by cGMP induction (Figure 2I). These data reinforce that ETEC are heterogenous in terms of ST production and in their responses to small molecules, including deferoxamine. 

### 3.2. Deferoxamine Reduces ST Production and Activity in CFA/I^+^ and CS6^+^ ST-ETEC Clinical Isolates While Changes in CF Expression Are Isolate-Dependent

**Figure 3:**
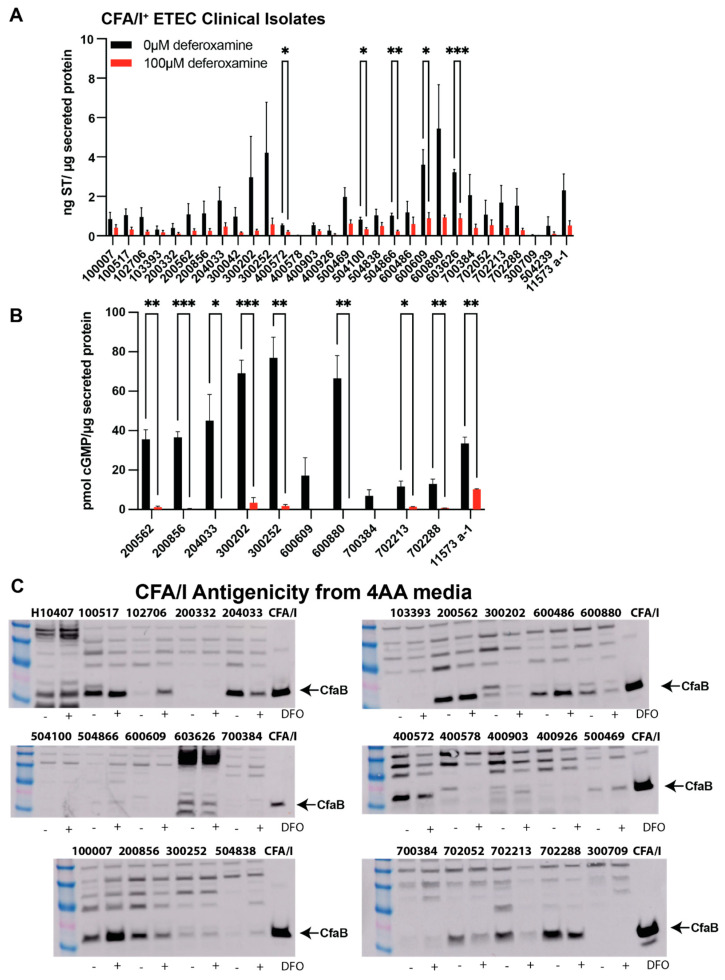
ST production and activity are decreased in CFA/I^+^ ST-ETEC isolates cultured with deferoxamine, while changes in CFA/I production are isolate-specific. Select ST-only CFA/I^+^-ETEC isolates from GEMS were cultured in chemically defined 4AA media with or without the addition of deferoxamine mesylate (100 µM) to induce iron limitation overnight before ST production (**A**), which was measured using the ST competitive ELISA. Broadly, addition of deferoxamine decreases ST production in ETEC supernatants. ETEC isolates were then down-selected based on ST production threshold of 2.0 ng/µg total protein, and supernatants (10 µg) were applied to T84 cells to determine ST-mediated induction of cGMP (**B**). Again, deferoxamine decreases ST activity measured from ETEC supernatants. Cell lysates from the same cultures demonstrate variable CFA/I production in the presence or absence of deferoxamine (**C**). The represented data in (**A**,**B**) represent the means of two individual experiments. The data represented in (**C**) was confirmed two times. Data were analyzed via T-tests within individual strains between culture conditions *, *p* < 0.05; **, *p* < 0.01; ***, *p* < 0.001.

We next screened a library of CFA/I^+^ clinical isolates obtained from cases of moderate-to-severe diarrhea from the GEMS study [6,14] for the impact of deferoxamine (100 µM) on CF expression and ST production/activity. ETEC isolates were cultured overnight in chemically defined 4AA media, which enhances ST production, and cell-free supernatants were screened for ST production and ST activity. Lysates were screened for CF expression. It is important to mention that the overnight culture OD for the isolates grown in the presence and absence of deferoxamine were similar. As shown for CFA/I^+^ ST-ETEC isolates, the inclusion of deferoxamine (100 µM) reduced ST production (Figure 3A) per total protein in all strains tested. In some strains, deferoxamine increased secreted protein levels. Next, we down-selected ETEC isolates based on an antigenicity threshold of 1.7–2 ng ST/µg total secreted protein to screen for ST toxicity. In short, 10 µg of cell-free supernatant from ETEC isolates grown in the absence and presence of deferoxamine were applied to confluent T84 cells for ST-mediated induction of cGMP. As shown, deferoxamine reduced ST activity per µg secreted protein in all ETEC isolates, although there was still measurable activity in ETEC strains 200562, 300202, 300252, 702052, 702213, and 11573 a-1 following deferoxamine treatment (Figure 3B); although the ST competitive ELISA identified ST in supernatants of ETC isolate 603626, application of these supernatants to T84 cells failed to induce cGMP. Next, we assessed CFA/I expression in ETEC lysates grown in the presence and absence of deferoxamine via Western blotting. As shown, CFA/I expression is not uniform across ETEC isolates (Figure 3C), consistent with different isolates having varying abilities to hemagglutinate red blood cells [24]. Similar to ETEC H10407, deferoxamine treatment increases CFA/I expression in some isolates (100007, 102706, 600486), but deferoxamine treatment also decreases CFA/I expression in other isolates (200856, 204033, 400572, 702052). Uncropped Western blots are available in the Supplementary Data. Together, these data reinforce the notion that CFA/I^+^ ETEC are heterogenous in their response to deferoxamine.

**Figure 4:**
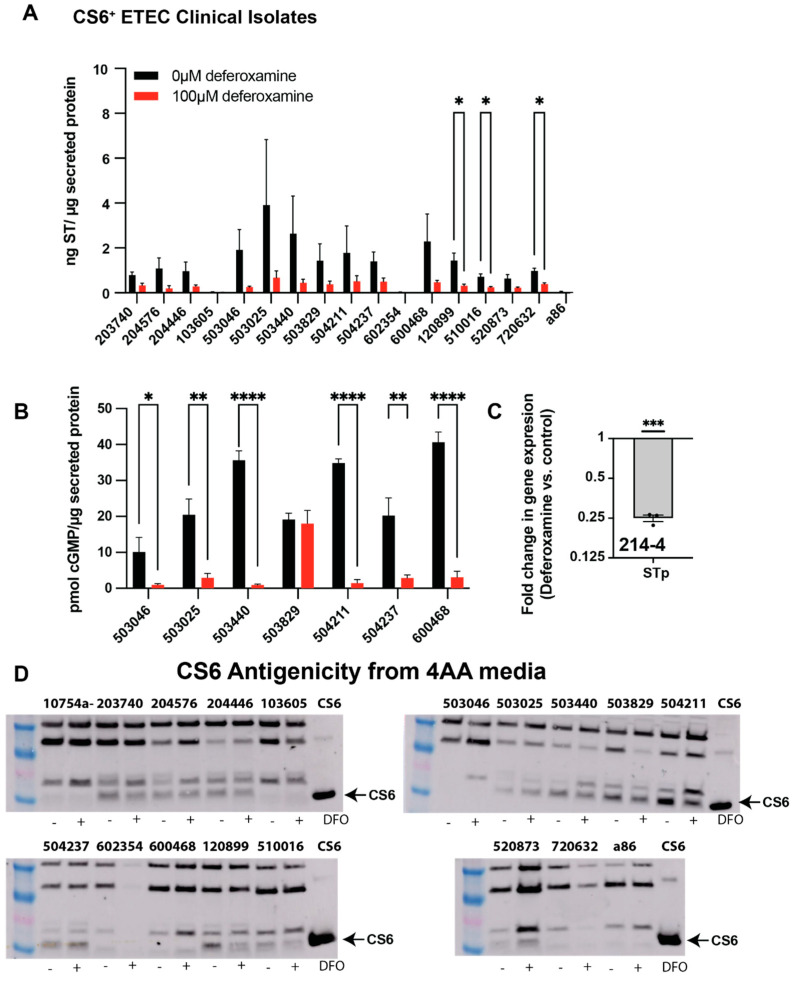
ST production and activity are decreased in CS6^+^ ST-ETEC isolates cultured with deferoxamine, while changes in CS6 production are isolate-specific. Select ST-only CS6^+^-ETEC isolates from GEMS were cultured in chemically defined 4AA media with or without the addition of deferoxamine mesylate (100 µM) to induce iron limitation overnight before ST production (**A**) was measured using the ST competitive ELISA. Broadly, addition of deferoxamine decreases ST production in CS6^+^-ETEC supernatants. ETEC isolates were then down-selected based on ST production threshold of 2.0 ng/µg total protein, and supernatants (10 µg) were applied to T84 cells to determine ST-mediated induction of cGMP (**B**). Again, deferoxamine decreases ST activity measured from ETEC supernatants. ETEC 214-4 was grown in chemically defined 4AA media with or without deferoxamine mesylate (100 µM) to mid-log phase (OD 0.5) before RNA isolation using the hot phenol/chloroform method. RNA was reverse transcribed into cDNA, which was used for qPCR to determine relative transcripts of genes expressing ST (porcine variant, STp, estP) as compared to genes expressing 16S rRNA. These qPCR data confirm that iron limitation via deferoxamine suppresses ST expression in ETEC 214-4 (**C**). Cell lysates from CS6^+^-ETEC isolates demonstrate variable CS6 production in the presence or absence of deferoxamine (**D**). The represented data in (**A**,**B**) represent the means of two individual experiments. The data represented in (**D**) was confirmed two times. Data in panels A and B were analyzed via T-tests within individual strains between culture conditions. Data in panel C were analyzed via a one-sample T-test against a hypothetical mean of 1. *, *p* < 0.05; **, *p* < 0.01; ***, *p* < 0.001; ****, *p* < 0.0001.

We next screened a library of CS6^+^ ST-ETEC clinical isolates obtained from cases of moderate-to-severe diarrhea from the GEMS study [6,14] for the impact of deferoxamine (100 µM) on CF expression and ST production/activity. Deferoxamine also decreases ST production per µg secreted protein in CS6^+^ ST-ETEC clinical isolates (Figure 4A). Furthermore, the ability to induce cGMP is broadly suppressed in many of the CS6^+^ ST-ETEC clinical isolates (Figure 4B). Interestingly, deferoxamine decreased ST production in ETEC isolate 503829 as measured via the ST competitive ELISA, and 503829 supernatants induced similar amounts of cGMP when applied to T84 cells. This could be explained by the fact that other antigenic ST isoforms exist and suggest that deferoxamine promotes the formation of the isoform that engages with the guanylyl cyclase C receptor to increase cGMP production. In support of the broad effects of deferoxamine on ST expression in diverse isolates, we demonstrate via qPCR that the gene encoding STp in ETEC 214-4 is significantly downregulated 4-fold following deferoxamine treatment (Figure 4C). Lysates from CS6^+^ ST-ETEC clinical isolates were then screened for CS6 antigenicity via Western blots (Figure 4D). Overall, we did not notice major impacts following the inclusion of deferoxamine, except we found that deferoxamine suppresses CS6 expression in ETEC isolates 503829, 504211, and 120899. Overall, these data demonstrated that deferoxamine suppresses ST production and activity, but no generalities could be made for changes in CFA/I or CS6 expression. 

### 3.3. Oral Deferoxamine Treatment Extends ETEC H10407 Colonization 

**Figure 5:**
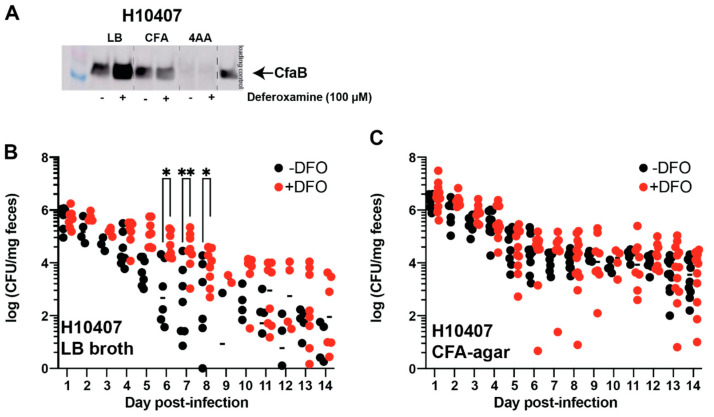
Oral deferoxamine pre-treatment extends duration of ETEC H10407 fecal shedding when grown in LB. ETEC are highly adaptable to environmental conditions, and culturing methods contribute to increased or decreased virulence. ETEC H10407 was cultured in LB broth, CFA agar, and 4AA broth overnight before preparation of cell lysates. CFA/I expression was measured by applying equal mass (10 µg) of lysates to gel for subsequent Western blot. As expected, CFA/I was expressed during all growth conditions but more highly expressed when grown in CFA medium (**A**). We also noted a 2.2-fold increase in CFA/I production when LB was supplemented with 100 µM deferoxamine, but not in the other media assessed. Thus, we cultured ETEC H10407 in LB broth (**B**) and on CFA agar (**C**) for assessment of the effect of iron limitation via deferoxamine on ETEC H10407 colonization as measured via daily fecal shedding. Interestingly, we observed that deferoxamine pre-treatment significantly prolonged the duration of ETEC H10407 fecal shedding in CD1 mice when initially grown in LB broth. Deferoxamine pre-treatment did not affect ETEC H10407 fecal shedding in CD1 mice when grown on CFA agar. Data were analyzed via two-way ANOVA with matched time points. *, *p* < 0.05; **, *p* < 0.01.

It is likely that the amount of cell-surface colonization factor steers initial ETEC pathogenesis in humans. Protocols that engender efficient ETEC H10407 colonization in controlled human infection models routinely use CFA agar to increase CFA/I production [24]. We confirm that the relative levels of CFA/I in ETEC H10407 lysates change when bacteria are cultured on CFA agar or in LB or 4AA broth (Figure 5A). As expected, CFA/I is most abundant when ETEC H10407 is cultured on CFA agar and least abundant when cultured in 4AA broth. This is not surprising since 4AA broth is a chemically defined medium that was optimized for ST production [25]. Western blot analysis showed that ETEC H10407 cultured in LB and deferoxamine (100 µM) caused a 2.20-fold increase in CFA/I production compared to LB alone. 

Although mice are not natural ETEC hosts, murine models can be used as a proxy to understand ETEC colonization and shedding in a mammalian host [16]. Here, we tested the impact of deferoxamine on ETEC H10407 shedding in a streptomycin mouse model [16]. Female CD1 mice (7–10 weeks old) were orally gavaged with deferoxamine (600 µg) 24 h before and immediately prior to ETEC inoculation. In this model, we show streptomycin causes a significant drop in fecal iron in all mice (Figure S1), but fecal iron levels in deferoxamine-treated mice and control mice were similar (data not shown). When ETEC H10407 was cultured in LB broth, oral deferoxamine treatment significantly increased ETEC fecal shedding on days 5–8 of infection (Figure 5B). This is consistent with the deferoxamine-mediated induction of CFA/I in ETEC H10407 when cultured in LB (Figure 5A). On the other hand, when ETEC H10407 was cultured on CFA agar, ETEC shedding rates were similar between mice treated with oral deferoxamine and control mice (Figure 5C). This supports CFA/I-mediated attachment to the intestinal epithelium since deferoxamine did not affect CFA/I production when grown on CFA agar, although the impact of iron restriction and deferoxamine on innate immune responses cannot be ruled out [38].

### 3.4. Oral Deferoxamine Treatment Shortens ETEC 214-4 Colonization 

**Figure 6:**
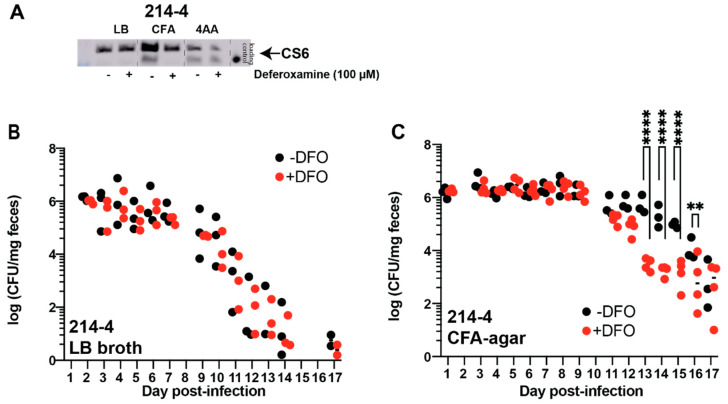
Oral deferoxamine pre-treatment quickens clearance of ETEC 214-4 via fecal shedding when grown in CFA agar. ETEC 214-4 was cultured in LB broth, CFA agar, and 4AA broth overnight before preparation of cell lysates. CS6 expression was measured by applying equal mass (10 µg) of lysates to gel for subsequent Western blot. CS6 was expressed during all growth conditions but more highly expressed when grown in CFA medium (**A**). Furthermore, a 25-fold decrease was noted in CS6 detected in CFA supplemented with 100 µM deferoxamine. Thus, we cultured ETEC 214-4 in LB broth (**B**) and on CFA agar (**C**) to assess the effect of iron limitation via deferoxamine on ETEC 214-4 colonization as measured via daily fecal shedding. Interestingly, we observed that deferoxamine pre-treatment did not affect fecal shedding rates of ETEC 214-4 when grown in LB broth. On the other hand, deferoxamine pre-treatment increased the rate at which ETEC 214-4 was cleared from CD1 mice, as measured by fecal shedding when grown on CFA agar. Data were analyzed via two-way ANOVA with matched time points. **, *p* < 0.01; ****, *p* < 0.0001.

Previous studies have shown that CS6 production is upregulated by iron and suppressed by iron restriction via deferoxamine [21]. Thus, we next assessed the role of deferoxamine on ETEC 214-4 colonization in the streptomycin mouse model. First, we cultured ETEC 214-4 in LB, CFA, and 4AA media and then supplemented each culture condition with exogenously added deferoxamine (100 µM). In contrast to the deferoxamine-mediated induction of CFA/I production shown for ETEC H10407, we demonstrate that deferoxamine suppresses CS6 production approximately 25-fold when cultured in CFA medium (Figure 6A). In contrast, when ETEC 214-4 was cultured in LB or 4AA, the addition of deferoxamine (100 µM) did not impact CS6 production, as compared to base media alone (Figure 6A). To demonstrate that ETEC growth conditions affect its ability to adhere to the intestinal epithelium and influence fecal shedding, we then cultured ETEC 214-4 in LB and CFA medium for in vivo assays. Mice were either left untreated or pre-treated with oral deferoxamine before ETEC 214-4 inoculation. As would be expected, oral deferoxamine pre-treatment did not affect ETEC 214-4 fecal shedding when it was cultured in LB (Figure 6B). We believe this to be because there were no changes to CS6 production between these culture conditions (Figure 6A). In contrast, when ETEC 214-4 was cultured on CFA agar, oral deferoxamine pre-treatment caused quicker clearance of the infection, with significant differences noted between groups on days 13-16 (Figure 6C). Again, this supports the in vitro data suggesting that deferoxamine suppresses CS6 production when grown in CFA (Figure 6A). 

As iron deficiency anemia is common in children in LMICs, these data suggest that ETEC may use chemical cues induced following enterotoxin activity, including changes to metal (sodium, magnesium, iron) and non-metal (chloride) ions to regulate intestinal colonization and pathogenesis. Further, based on our understanding of colonization factor and toxin production in diverse ETEC isolates, these data support future studies to elucidate the molecular mechanisms associated with the broadly divergent responses to iron and iron limitation seen in CFA/I- and CS6-ETEC isolates. 

## 4. Discussion

This study is important because it demonstrates that enterotoxin activity alters the luminal concentrations of metal and non-metal ions that may signal to ETEC to alter virulence gene expression in the intestinal microenvironment. To our knowledge, this is the first study to profile the kinetics of multi-ion flux in luminal secretions using ICP-MS following oral enterotoxin exposure. This is a unique and innovative approach that can be further adapted to understand the role of host ion flux during ETEC disease pathogenesis.

Ussing chambers have been used to establish that ST causes chloride secretion across intact epithelial monolayers; thus, we reasoned that ICP-MS could be used to further examine the flux of chloride and metal and non-metal ions following oral ST intoxication. We screened luminal secretions for chloride based on the expectation that chloride would be elevated during times we saw increased secretion. As expected, we identified a transient increase in luminal chloride following oral ST intoxication. We also demonstrate that sodium chloride suppresses CFA/I expression in ETEC H10407, suggesting that increased luminal chloride serves to enhance the dissemination of ETEC H10407 by decreasing its adherence to the intestinal epithelium. On the other hand, however, we also found that oral ST intoxication decreases luminal magnesium concentrations transiently. We further demonstrate that magnesium restriction promotes CFA/I expression. ST-mediated decreases in luminal magnesium could be a compensatory mechanism that enables ETEC CFA/I production during times of enhanced enterotoxin activity. Recent studies have demonstrated that low magnesium levels activate the PhoP/PhoQ two-component regulatory system in enterohemorrhagic *E. coli* to enhance attachment and effacement via activation of the LEE loci [39]. However, the opposing impacts of increased chloride and decreased magnesium on CFA/I expression suggest that there may be compensatory mechanisms regulating ETEC adherence to the host epithelium. Previous studies have identified causal relationships between magnesium and chloride ions. It could be assumed that decreased luminal magnesium bioavailability results in either increased intracellular or serosal magnesium. Increased magnesium concentrations on the serosal side of intact rabbit ileal epithelium result in the unidirectional flux of chloride from the serosa to the mucosa [40], essentially functioning as an osmotic laxative. Indeed, magnesium citrate is an over-the-counter medication used to provide short-term constipation relief. Elevated extracellular calcium opposes cellular proliferation via the activation of calcium-sensing receptors (CaRs), and ST-mediated stimulation of the GC-C receptor recruits CaRs to the apical membrane [41]. Thus, increased luminal calcium may be required for the anti-proliferative effects of ST [42]. Further ion-mediated outcomes on ST and CFA/I expression will require future investigations to resolve pathogenic mechanisms employed by ETEC.

Other than secretion, ETEC are associated with increased morbidity, which is almost assuredly associated with repeated enterotoxin activity. Children can be infected with similar strains of ETEC without developing long-term protective immunity since antibodies to LT alone do not mediate protection [43], suggesting that ETEC have evolved mechanisms to subvert the development of mucosal immunity. Recent studies have shown that patients with a point mutation in the transferrin receptor have combined B and T cell immunodeficiency characterized by iron deficiency [44]. We have previously shown that ETEC’s enterotoxins induce IL-33 production in small intestinal mucosal scrapings following oral enterotoxin exposure [23,27]. IL-33 is an epithelial cytokine involved in both physiological and immune responses in mucosal tissues, where it promotes anemia during chronic inflammation by decreasing hemoglobin expression and inhibiting the differentiation of erythroid progenitors during erythropoiesis [45]. Further, IL-33 and hemin are required for the differentiation of blood monocytes into splenic red pulp macrophages that function to recycle iron from senescent erythrocytes [46]. These data are also underscored by the fact that childhood anemia is highest in areas of the world in which ETEC is endemic, suggesting that ETEC could modulate host iron homeostasis and lead to increased morbidity.

Enterotoxins can provide the expressing organisms a competitive advantage over non-expressing organisms for iron and long-chained fatty acid acquisition [47]. Our previous transcriptional studies demonstrated that ST intoxication of intestinal epithelial cells resulted in an overall similarity to the deferoxamine-mediated transcriptional programming of intestinal epithelial cells [18,36]. Deferoxamine mesylate is a commonly used siderophore that induces IL-8 production in HT29, Caco-2, and T84 intestinal epithelial cells via mitogen-activated protein kinase activity [36]. Likewise, ST induces IL-8 production from intestinal epithelial cells, and we previously demonstrated that ST is capable of binding iron and zinc under reducing conditions [18], although whether ST functions as a metal-binding peptide in vivo is unknown. Further, it is well-known that Na^+^/H^+^ exchangers are important modulators of ST-mediated secretory diarrhea, and the inhibition of Na^+^/H^+^ exchangers abolishes deferoxamine-mediated IL-8 production [36]. Together these data suggest that ST activity could modulate iron metabolism and polarize mucosal immune responses.

Here, we demonstrate that iron limitation via deferoxamine suppresses ST production/secretion in diverse ST-only CFA/I^+^- and CS6^+^-ETEC isolates [14], linking iron availability to ST production and activity [18]. Iron-containing transcription factors, including FNR and IscR, have been implicated in attachment phenotypes in ETEC H10407 [20,48]. Using the streptomycin mouse model, we also demonstrate that two-day oral deferoxamine treatment increased the duration of ETEC H10407 fecal shedding when the inoculum was grown in LB broth but not when the inoculum was grown on CFA agar. This is in line with deferoxamine-mediated increases in CFA/I production in ETEC H10407 [20] but also suggests that CFA/I expression may reach an upper limit when grown on CFA agar. On the other hand, deferoxamine does not affect the fecal shedding of ETEC 214-4 when grown in LB, but deferoxamine pre-treatment significantly shortens the fecal shedding of ETEC 214-4 when grown on CFA agar. This supports previous studies that demonstrated CS6 is induced by iron supplementation. Combined, these data suggest that iron limitation induces CFA/I production in ETEC H10407, but excess iron induces CS6 production in ETEC 214-4. Epidemiologically, more than half of all wild ETEC isolates lack a recognizable colonization factor [6], suggesting that other adhesins, including the type 1 fimbriae, FimH, may be important in regulating intestinal colonization [49].

Future in vivo investigations of CFA/I^+^ and CS6^+^ ETEC cultured in iron-limiting conditions could provide valuable information regarding ETEC colonization [50]. The differential regulation of colonization factors and ST in response to iron limitation is in line with the prevailing belief that enterotoxins increase the dissemination of the expressing organisms; however, a full understanding of colonization factor and toxin transcriptional responses in these isolates remains to be fully investigated. 

Together, these data demonstrate that enterotoxin-liberated ions modulate ETEC pathogenesis. More broadly, these data demonstrate that ETEC strains must be considered individually when assessing the environmental conditions that lead to diarrheal disease. Future studies will be required to understand overlapping and targetable regulons for ameliorating secretory diarrheal disease and understand how luminal ion concentrations affect host and pathogen responses. 

One limitation of this study is that we did not assess the impacts of LT on changes in luminal ions/metals either alone or in combination with ST. This is important because the evolutionary rationale for many ETEC isolates to carry both enterotoxins (ST and LT) when either alone is sufficient to cause diarrheal disease still evades understanding. Recent studies have demonstrated that the transcriptional activity of LT is repressed via cAMP receptor protein (CRP), while the transcriptional activity of ST is enhanced by CRP [51], suggesting that in vivo LT and ST expression could be regulated by metal and non-metal ions and the postprandial flux of glucose availability.

Given the high burden of disease due to ST-ETEC [6,52], the findings in this study will steer future directions aimed at understanding differences in the regulation of virulence in most pathogenic isolates as compared to the least pathogenic isolates. Potentially, a nuanced understanding of luminal ion flux will also help define how some ETEC isolates remain asymptomatic in the host. Furthermore, this study underscores the need to develop more refined models of ETEC pathogenesis since the application of strategies discovered from an understanding of the most severe ETEC isolates may not translate to the majority of isolates. 

## 5. Conclusions

Certain electrolytes, including chloride and sodium, are increased in the intestinal lumen following enterotoxin activity. Here, we demonstrate that ST intoxication in mice causes changes in the luminal concentrations of chloride, magnesium, iron, and calcium. However, how these changes in ion concentrations impact ETEC pathogenesis, including colonization factor expression of toxin expression, remains a gap in knowledge. As expected, ST activity causes a transient increase in luminal chloride, and we show that increases in luminal chloride (via NaCl) suppress CFA/I expression in ETEC H10407. ST activity causes a transient decrease in luminal magnesium, and we show that decreased magnesium induces CFA/I expression in ETEC H10407. Thus, we analyzed a library of ETEC isolates for colonization factor expression and ST expression in the presence and absence of deferoxamine, an iron chelator. Lastly, using a streptomycin mouse model, we demonstrate that deferoxamine extends ETEC H10407 fecal shedding when grown in LB but not CFA agar. Combined, these data suggest that ETEC are finely adapted to the environment and that ion-mediated impacts on one strain may not be applicable to others.

## Data Availability

The data that support the findings of this study are available in the methods and/or available from the corresponding author on request.

## Supplementary Materials

The following supporting information can be downloaded at: https://www.mdpi.com/article/10.3390/cells12040567/s1. Figure S1: Oral streptomycin treatment significantly decreases fecal iron levels in female CD1 mice. Table S1: ETEC isolates used in this study. Table S2: Primers used in this study.

## Institutional Care and Use Committee

All experiments were approved by the Institutional Care and Use Committee at the Tulane University School of Medicine.
