# Supplementary material for: Heat-Stable Enterotoxin Secretions Assessed via ICP-MS Reveal Iron-Mediated Regulation of Virulence in CFA/I- and CS6-Expressing ETEC Isolates
Source: Cells. 2023 Feb 10;12(4):567. doi: 10.3390/cells12040567 (PMC9954033; doi:10.3390/cells12040567)
Supplement: Supplementary file 1 [file cells-12-00567-s001.zip › cells-2053121-SI.pdf]

Supplementary Materials: Figure S1: Oral streptomycin treatment significantly decreases fecal iron levels in female CD1 mice. Table S1: ETEC isolates used in this study. Table S2: Primers used in this study.

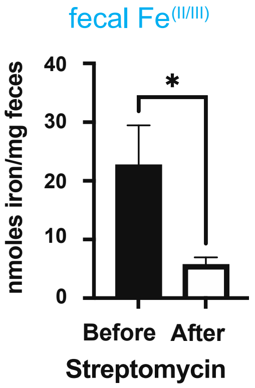

Figure S1. Streptomycin treatment reduces fecal iron in CD1 mice. Fecal pellets were collected before and after streptomycin treatment and incinerated, then the ashes were dissolved, and iron content tested via ferrozine assay. Data were analyzed via t-test. *, $p < 0.05$.

Supplemental Table S1. Bacterial strains used in this study

Strains /Plasmid	Major characteristics	Reference
H10407	CFA/I, STh, STp, LT (Bangladesh)	[10]
214-4	CS6$^+$, STp$^+$ (Mexico)	[11]
100007	CFA/I, CS21, STh (Gambia)	[6]
100517	CFA/I, CS21, STh (Gambia)	[6]
102706	CFA/I, STh (Gambia)	[6]
103393	CFA/I, CS21, STh (Gambia)	[6]
200332	CFA/I, CS21, STh (Mali)	[6]
200562	CFA/I, CS21, STh (Mali)	[6]
200856	CFA/I, CS21, STh (Mali)	[6]
204033	CFA/I, CS21, STh (Mali)	[6]
300042	CFA/I, CS21, STh (Mozambique)	[6]
300202	CFA/I, CS21, STh (Mozambique)	[6]
300252	CFA/I, CS21, STh (Mozambique)	[6]
302005	CFA/I, CS21, STh (Mozambique)	[6]
400572	CFA/I, CS21, STh (Kenya)	[6]
400578	CFA/I, CS21, STh (Kenya)	[6]
400903	CFA/I, STh (Kenya)	[6]
400926	CFA/I, CS21, STh (Kenya)	[6]
500469	CFA/I, CS21, STh (India)	[6]
504100	CFA/I, CS21, STh (India)	[6]
504838	CFA/I, CS21, STh (India)	[6]
504866	CFA/I, CS21, STh (India)	[6]
600486	CFA/I, CS21, STh (Bangladesh)	[6]
600609	CFA/I, CS21, STh (Bangladesh)	[6]
600880	CFA/I, CS21, STh (Bangladesh)	[6]

603626	CFA/I, CS21, STh (Bangladesh)	[6]
700384	CFA/I, CS21, STh (Pakistan)	[6]
702052	CFA/I*, CS21, ST* (Pakistan)	[6]
702213	CFA/I, CS21, STh (Pakistan)	[6]
702288	CFA/I, CS21, STh (Pakistan)	[6]
300307	CFA/I, CS21, STh (Mozambique)	[6]
504239	CFA/I, CS21, STh (India)	[6]
11573 a-1	CFA/I, CS21, STh (Chile)	[53]
10802 a	CFA/I, CS21, STh (Chile)	[53]
10754 a-1	CFA/I, CS21, STh (Chile)	[53]
203740	CS6, STh (Mali)	[6]
204576	CS6, CS5, STh (Mali)	[6]
204446	CS6, CS5, STh (Mali)	[6]
103605	CS6, CS5, STh (Gambia)	[6]
503046	CS6, CS4, CS21, STh (India)	[6]
503025	CS6, CS5, STh (India)	[6]
503440	CS6, CS21, STh (India)	[6]
503829	CS6, CS21, STh (India)	[6]
504211	CS6, CS21, STh (India)	[6]
504237	CS6, CS5, STh (India)	[6]
602354	CS6, CS5, STh (Bangladesh)	[6]
600468	CS6, STh (Bangladesh)	[6]
120899	CS6, CS5, STh (Gambia)	[6]
510016	CS6, CS4, CS21, STh (India)	[6]
520873	CS6, CS4, CS21, STh (India)	[6]
720632	CS6, STh (Pakistan)	[6]
a86	CS6, STh (Chile)	[53]
ETEC H10407-Sp	ETEC H10407, Str^r	This study
ETEC 214-4-Sp	ETEC 214-4, Str^r	This study

Note: Str^r for streptomycin resistance

Supplemental Table S2. qPCR primers used in this study

Description	Sequence (5`-3`)
cfaB Fw	GAGTGCTTCAGCAGTAGAGAAA
cfaB Rev	TGATGCGGGAGAATAAGCTAAC
cfaE Fw	ACAGCAGCTCTTTGGAGATAAG
cfaE Rev	TGCCAGGAGAAGTGACAAAG
16S rRNA Fw	GAAGGCTACGATCTCGGTAAAG
16S rRNA Rev	GGTACGAGACGTTCCTTGATAC
estP Fw	AGCTAATGTTGGCAA
estP Rev	ACAAGCAGGATTACAACA
fepA Fw	TTCATTCCCTGGCCTTGTTG

fepA Rev	CAGGCGCCTGTAAGTTCT
cssA Fw	CGGCAGCCATGCCAGAAC
cssA Rev	TGGATTTGTGGTTCAGGCGC
cssB Fw	CCGGAGTGGTAAATGCAGGA
cssB Rev	AGGGATCGTCATCTCAACCG
